# Editorial: Biology of Cognitive Aging: Model Systems, Technologies, and Beyond

**DOI:** 10.3389/fgene.2015.00366

**Published:** 2016-01-12

**Authors:** Shin Murakami

**Affiliations:** Department of Basic Sciences, College of Osteopathic Medicine, Touro University-CaliforniaVallejo, CA, USA

**Keywords:** research outreach, Alzheimer's disease, Huntington disease, dementia, age-related memory impairment (AMI), age-related memory loss, model systems, patient care

Age-related memory impairment (AMI), also called age-associated memory impairment (AAMI), describes the normal emergence and progression of memory problems associated with aging. It is one of diverse age-associated phenotypes found across a variety of organisms, including bees, fruit flies, nematodes, snails, rodents, and humans. The goal of this editorial is to provide an overview of our current understanding of AMI and its progression to more severe deficits related to advanced age and dementia. This research issue focuses on model systems and technologies relevant to cognitive aging and Alzheimer's disease, and the study of AMI using invertebrate disease models.

AMI can be viewed as a reduction in neuroplasticity, wherein the retention of existing memories is relatively well-preserved, compared with the ability to acquire new information. Aging specifically compromises mental processes underlying learning and some types of long-term memory, while leaving other types of long-term and procedural memories intact. AMI, however, may also include loss of existing memory, a condition termed age-related memory loss. In a simplified model, cognitive aging can be described to occur in the following sequence: AMI (normal state), mild cognitive impairment (MCI [transition state]), and then lastly, dementia (disease state). The neural mechanisms underlying the development of AMI into more advanced age-related disease remain unclear.

A wide variety of model systems and comparative analysis techniques show promise in guiding therapeutic strategies for elderly patients with memory impairments. Despite their potential, the use of disease models in this field of medicine has been discouraged. As stated by the Alzheimer's Association^1^, “a limitation of these models is that they do not capture the full complexity of the human condition, which is problematic if one wants to use them to predict the success of specific therapeutic interventions in individuals with Alzheimer's disease (Accessed on Dec 2015).” This research issue thoroughly discusses both the advantages and limitations of disease models of cognitive aging.

A major aim of this editorial is to compile information about and identify connections between patients with dementia and other organisms, such as bees (Farooqui), fruit flies (Mukherjee et al.), nematodes (Alexander et al.; Gkikas et al.; Machino et al.; Scerbak et al.; Therrien and Parker), snails (Hermann et al.), fish (Newman et al.), and rodents (Webster et al.; Way et al.); a manuscript co-authored by an early-stage patient with Alzheimer's disease (Murakami and Halperin) is also discussed. The work described herein includes rigorous reviews and original research articles on models of Alzheimer's disease, Parkinson's disease, Huntington's disease, Amyotrophic lateral sclerosis, and other neurodegenerative conditions. Alexander et al. comprehensively review neurodegenerative disease in the nematode, *Caenorhabditis elegans* (*C. elegans*), while Webster et al. address the significance of mouse models to our understanding of human Alzheimer's disease. Gkikas et al. discuss pathways of longevity that potentially restore memory function during aging in *C. elegans*. Machino et al. propose the use of a semi-automated motion-tracking system as a platform for genetic and pharmacological screening, which was inspired by feedback from an early-stage patient with Alzheimer's disease (Murakami and Halperin). The study of AMI and dementia also utilizes other non-mammalian models, such as the freshwater snail (Hermann et al.) and a zebra fish model of Alzheimer's disease (Newman et al.), among others.

Animal models enable functional analysis of AMI and dementia and provide useful information for minimizing the risks for humans. Thus, it would not be beneficial to underestimate the role of model systems in medicine. Additionally, research using such systems would be better positioned to advance future therapies if patient feedback is incorporated. To this end, Murakami and Halperin have proposed soliciting feedback from patients in an effort to align basic research with clinical practice, patient care, and the patient experience. Such feedback emphasizes the importance of existing comorbidities and potential interaction/association between co-occurring diseases. This approach is consistent with the “mid-life crisis theory,” in which earlier deleterious events are associated with aging and later age-related diseases (Murakami et al., 2011; Murakami, 2013).

I thank all of the authors for their contributions. I would especially like to thank my friend and collaborator, Dr. Alexander “Sandy” Halperin, who, despite difficult personal circumstances, continues to provide valuable insight from a patient's point of view.

## Author contributions

SM conceived and wrote this editorial.

### Conflict of interest statement

The author declares that the research was conducted in the absence of any commercial or financial relationships that could be construed as a potential conflict of interest.

## Footnotes

^1^Alzheimer's association. *Areas of Focus for the 2014 International Research Grant Program*. Available online at: http://www.alz.org/research/downloads/2014_area_of_focus.pdf (Accessed on Dec 2015).
